# Does body mass index early in pregnancy influence the risk of maternal anaemia? An observational study in Indonesian and Ghanaian women

**DOI:** 10.1186/s12889-018-5704-2

**Published:** 2018-07-13

**Authors:** Martina Mocking, Ary I. Savitri, Cuno S. P. M. Uiterwaal, Dwirani Amelia, Edward Antwi, Mohammad Baharuddin, Diederick E. Grobbee, Kerstin Klipstein-Grobusch, Joyce L. Browne

**Affiliations:** 1 Julius Global Health, Julius Center for Health Sciences and Primary Care, University Medical Center Utrecht, Utrecht, The Netherlands; 2 Budi Kemuliaan Hospital, Jakarta, Indonesia; 3 Ghana Health Service, Accra, Ghana; 4 Division of Epidemiology and Biostatistics, School of Public Health, Faculty of Health Sciences, University of the Witwatersrand, Johannesburg, South Africa

**Keywords:** LMIC’s, Early pregnancy BMI, Anaemia

## Abstract

**Background:**

Anaemia is common among pregnant women, especially in low- and middle-income countries (LMICs). While body mass index (BMI) relates to many risk factors for anaemia in pregnancy, little is known about the direct relation with anaemia itself. This is particularly relevant in Southeast Asia and Sub-Saharan Africa where the prevalence of anaemia in pregnancy and the associated adverse outcomes is among the highest worldwide. This study aimed to assess the association between early pregnancy BMI and anaemia at first antenatal care visit in Indonesian and Ghanaian women. In addition, the associations between early pregnancy anaemia and adverse birth outcomes was assessed.

**Methods:**

Prospective cohort studies of women in early pregnancy were conducted in Jakarta, Indonesia (*n* = 433) and in Accra, Ghana (*n* = 946), between 2012 and 2014. Linear regression analysis was used to assess relations between early pregnancy BMI and pregnancy haemoglobin levels at booking. Logistic regression analyses were used to assess associations between early pregnancy anaemia as defined by the World Health Organization (WHO) criteria and a composite of adverse birth outcomes including stillbirth, low birth weight and preterm birth.

**Results:**

Indonesian women had lower BMI than Ghanaian women (23.0 vs 25.4 kg/m^2^, *p* < 0.001) and higher mean haemoglobin levels (12.4 vs 11.1 g/dL, *p* < 0.001), corresponding to anaemia prevalence of 10 and 44%, respectively. Higher early pregnancy BMI was associated with higher haemoglobin levels in Indonesian (0.054 g/dL/kg/m^2^, 95% CI 0.03 to 0.08, *p* < 0.001) and Ghanaian women (0.044 g/dL/kg/m^2^, 0.02 to 0.07, *p* < 0.001). Accordingly, risk for anaemia decreased with higher early pregnancy BMI for Indonesians (adjusted OR 0.88, 0.81 to 0.97, *p* = 0.01) and Ghanaians (adjusted OR 0.95, 0.92 to 0.98, *p* < 0.001). No association between anaemia and the composite of adverse birth outcomes was observed.

**Conclusion:**

Higher BMI in early pregnancy is associated with higher haemoglobin levels at antenatal booking and with a reduced risk of anaemia in Indonesian and Ghanaian women.

**Electronic supplementary material:**

The online version of this article (10.1186/s12889-018-5704-2) contains supplementary material, which is available to authorized users.

## Background

Anaemia is a major public health problem affecting 1.62 billion people globally [1]. Notably, 32 million (38%) pregnant women are anaemic, of whom 750,000 severely anaemic (haemoglobin level < 7.0 g/dL) [2]. In low- and middle-income countries (LMICs) maternal anaemia occurs in 43% of pregnant women, with the highest prevalence found in Southern Asian (52%), Central African and West African countries (56%) [3].

Anaemia in pregnancy, particularly in LMIC, is associated with lower socioeconomic status [1], young maternal age, low parity [1, 4, 5], ethnicity [6, 7], being in the third trimester [4, 7–9], and having many children [1, 7, 9]. Causes include micronutrient deficiencies (including iron, vitamin B12, folic acid and vitamin A), parasitic infections (such as malaria, helminth, hookworm and schistosomiasis), HIV/AIDS, chronic inflammation and genetic haemoglobinopathies (e.g. thalassaemia) [1, 3, 10]. Anaemia in pregnancy is associated with increased perinatal and maternal mortality [11], and other adverse outcomes including low birth weight, preterm birth [1, 3, 6, 10, 12] and reduced maternal productivity due to fatigue [1, 13].

There is a growing interest in the pre- and early pregnancy maternal physical condition, particularly body mass index (BMI), and its role in healthy pregnancy [14–17]. Underweight in (early) pregnancy is associated with preterm birth, low birthweight, miscarriage and having a small for gestational age baby [14, 18–20], whilst protecting against hypertensive disorders of pregnancy and gestational diabetes [14, 17, 18]. In contrast, maternal overweight and obesity are associated with higher risks for adverse obstetric and perinatal outcomes, including delivery complications, hypertensive disorders of pregnancy, gestational diabetes, macrosomia, and stillbirth [21–24].

In many South Asian and West and Central African countries, anaemia in pregnancy, persistent underweight and simultaneously increase in prevalence of overweight and obesity are common and potentially related health problems. A number of studies reported that low BMI increases the risk of anaemia in pregnancy [8, 14, 18]. However, these studies were limited by methodological choices such as uncertain anaemia cut-off levels or varying gestational ages at which haemoglobin level was determined [25]. Using cohorts from Indonesia and Ghana, in the present study we assessed whether early pregnancy BMI is related to anaemia at first antenatal booking.

## Methods

### Study design and study population

This study was conducted using data from two prospective cohort studies from Indonesia and Ghana, previously described in detail [26, 27].

#### Indonesian cohort

From February 2013 to October 2014 pregnant women were recruited during their first antenatal care visit at the private mother–child health Budi Kemuliaan Hospital and its branch Budi Kemuliaan Petojo. There were no particular inclusion criteria and routine antenatal blood tests were performed at booking in all recruited women with gestational age lower than 18 weeks. After enrolment, women were seen during their subsequent antenatal care (ANC) visits and followed up until delivery. Information was obtained about women’s socioeconomic status (level of education and current employment), current pregnancy (last menstrual period to determine gestational age and early pregnancy weight) and obstetrical history (gravidity). To estimate exposure to secondhand smoking, the spouse’s smoking status was documented.

#### Ghanaian cohort

From July 2012 to March 2014, 1010 pregnant women from the Accra Metropolis in Ghana were enrolled at the Ghana Health Services facilities Maamobi General Hospital and Ridge Hospital’s outpatient clinic. Women were eligible for participation if they were less than 17 weeks pregnant, 18 years or older and did not have a history of hypertension or hypertension at booking. Information was obtained on socio-demographic characteristics, socioeconomic status (level of education and economic activity) and health status including obstetrical status and history. Women were followed up until six weeks postpartum. Information about smoking was not recorded given the low prevalence of smoking in this population (< 0.3%) [28].

### Data set, entry and validation

For both cohorts, analyses were restricted to women with available early pregnancy BMI and early pregnancy blood test results. Missing data was not imputed because of low occurrence and complete cases were analysed.

### Predictors

Early pregnancy BMI was calculated as weight (in kilograms) divided by the square of height (in meters) [2], in which Indonesian women’s weight was based on the self-reported pre-pregnancy weight. For Ghanaian women, maternal weight at booking was used as early pregnancy weight.

### Outcomes

#### Primary outcome

Haemoglobin concentration and anaemia status were measured as primary outcomes. Anaemia was defined as a haemoglobin concentration below 11.0 g/dL, according to the WHO’s definition for anaemia in pregnancy [29].

Haemoglobin levels were measured in accordance with routine antenatal care standards by local laboratories.

#### Secondary outcome

As a secondary outcome, a composite of adverse birth outcomes was created and included stillbirth, low birth weight and preterm birth. Stillbirth was defined as the death of the foetus after 24 weeks of gestation and low birth weight was defined as birth weight < 2.500 g. Preterm birth was defined as birth at less than 37 weeks of gestation. Gestational age was determined by last menstrual period (in Indonesian cohort) or by ultrasound at or before the first antenatal care visit (in Ghanaian cohort) [30].

### Confounders

A priori, the following variables were considered as possible confounders of the relationship between early pregnancy BMI and haemoglobin level or anaemia: maternal age, gravidity, employment status and highest completed education [1, 12, 17, 24]. Employment status was classified as not working, working in a formal job or working in a non-formal job (trader, nanny, cleaning service, etc). Education was categorized into low (including uneducated, completed elementary school, and completed junior high school), intermediate (completed senior high school), and high (university degree or professional education).

Furthermore, secondhand smoking exposure was considered as potential confounder [2, 18, 29]. and was adjusted for in the Indonesian cohort. Comorbidities associated with anaemia, including sickle cell disease, glucose-6-phosphate dehydrogenase (G6PD) deficiency and malaria, were also considered as confounders [31, 32] and were adjusted for in the Ghanaian cohort.

### Data analysis

Women’s baseline characteristics were tabulated by their early pregnancy BMI. Due to major dissimilarities between Ghanaian and Indonesian BMI distribution, we used tertiles instead of World Health Organisation (WHO) BMI categories. Moreover, controversy exists on applying the existing WHO BMI categories for Asian populations, in which previous studies suggested lower cut-offs [33, 34]. Differences between tertiles of early pregnancy BMI were statistically tested using one-way ANOVA or Kruskal-Wallis for continuous and Pearson’s Chi-square or Fisher’s exact test for categorical variables, where appropriate.

Linear regression analysis was used with haemoglobin concentration as the dependent and early pregnancy BMI as the independent continuous variable. Logistic regression analysis was used with anaemia (yes/no) as the dependent and early pregnancy BMI as the independent continuous variable. For both types of analysis, we also separately evaluated the effect of weight and height on the outcomes. Logistic regression was also used to assess relations between anaemia and composite adverse birth outcomes. Results are expressed as linear regression coefficients or odds ratios and 95% confidence intervals. A *p*-value < 0.05 was considered statistically significant. All analyses were performed using IBM SPSS Statistics (version 21.0 for Windows) and were reported in accordance to STROBE Statement [35].

### Study approval

For the Indonesian cohort ethical approval was granted by the Institutional Review Board of Budi Kemuliaan Hospital. For the Ghanaian study population approval was obtained from Ghana Health Services Ethical Review Committee (GHS-ERC 07–9-11). In both cohorts, participating women provided written or thumbprint informed consent prior to study enrolment.

## Results

Of the Indonesian cohort, 433 women were included in the present analysis and 946 women from the Ghanaian cohort. Table 1 shows the baseline characteristics of the women according to their early pregnancy BMI tertiles, Additional file 1: Table S1 provides an overview of missing data.

**Table 1:** Baseline characteristics of the study populations categorized by early pregnancy BMI tertiles

Early pregnancy BMI	Indonesian cohort			*P* value	Ghanaian cohort			*P* value
	Lower tertile (*n* = 145)	Middle tertile (*n* = 143)	Upper tertile (*n* = 148)		Lower tertile (*n* = 334)	Middle tertile (*n* = 329)	Upper tertile (*n* = 336)	
BMI tertile range	12.5, 20.6	20.7, 24.3	24.4, 39.2		15.4, 23.1	23.1, 27.0	27.1, 42.3	
*Maternal characteristics*								
Age (y)	26.5 (6.06)	29.1 (5.18)	30.2 (6.06)	< 0.0011	26.3 (4.5)	28.0 (5.3)	29.7 (5.0)	< 0.0011
Height (cm)	156.7 (5.7)	155.8 (5.9)	155.4 (5.8)	0.151	161.4 (6.4)	161.3 (6.6)	160.5 (6.4)	0.141
Weight (kg)	45.6 (4.5)	54.7 (5.0)	67.5 (8.6)	< 0.0011	53.8 (6.1)	64.7 (6.2)	79.1 (9.4)	< 0.0011
Education (%)				0.792				0.372
*low*	25 (17.4)	22 (15.4)	25 (16.9)		88 (26.3)	83 (25.2)	69 (20.5)	
*medium*	94 (65.3)	90 (62.9)	89 (60.1)		214 (64.1)	206 (62.6)	223 (66.4)	
*high*	25 (17.4)	31 (21.7)	34 (23.0)		32 (9.6)	40 (12.2)	44 (13.1)	
Employment (%)				0.022				0.262
*not working*	67 (46.5)	66 (47.1)	87 (58.8)		47 (14.1)	50 (15.2)	35 (10.4)	
*non-formal*	8 (5.6)	13 (9.3)	17 (11.5)		245 (73.4)	237 (72.0)	265 (78.9)	
*formal*	69 (47.9)	61 (43.6)	44 (29.7)		42 (12.6)	42 (12.8)	36 (10.7)	
Second hand smoking (%)	31 (57.4)	34 (66.7)	35 (56.5)	0.492				
*Obstetric characteristics*								
Gestational age (wk)^a^	9.1 (3.4)	8.7 (3.5)	9.4 (3.3)	0.143	12.2 (2.9)	12.3 (2.8)	11.7 (3.0)	0.113
Gravidity (%)				< 0.0012				< 0.0012
*primigravida*	67 (57.8)	39 (34.5)	34 (27.0)		99 (29.6)	80 (24.3)	44 (13.1)	
*multigravida*	49 (42.2)	74 (65.5)	92 (73.0)		235 (70.4)	249 (75.7)	292 (86.9)	
*Clinical characteristics*								
Haemoglobin (g/dL)	12.0 (1.3)	12.4 (1.1)	12.6 (1.0)	< 0.0011	10.9 (1.8)	11.1 (1.4)	11.5 (1.5)	< 0.0011
MCV below 80.0 fL (%)	42 (29.0)	27 (19.0)	29 (19.9)	0.082				
Comorbidities^b^					56 (16.8)	52 (15.8)	46 (13.7)	0.532
*Birth outcomes*								
Low birth weight (%)	7 (6.4)	5 (4.5)	5 (4.0)	0.682	22 (8.8)	20 (7.7)	16 (6.0)	0.492
Prematurity (%)	5 (4.5)	5 (4.5)	7 (5.7)	0.892	18 (7.4)	22 (8.9)	20 (8.0)	0.832
Stillbirth (%)	1 (0.9)	1 (0.9)	1 (0.8)	1.004	3 (1.2)	2 (0.8)	1 (0.4)	0.464

Significant at *P* value< 0.005; 1 One-way ANOVA; 2 Chi-square; 3 Kruskal-Wallis; 4 Fisher’s exact test with *N* < 5

^a^at first ANC visit and based upon last menstrual period or ultrasound

^b^red blood cell affecting comorbidities including sickle cell disease, G6PD deficiency and malaria infection

The BMI of the first, second, and third tertile ranged between 12.5 to 20.6, 20.6 to 24.3 and 24.4 to 39.2 kg/m^2^ for Indonesian women and 15.4 to 23.1, 23.1 to 27.0 and 27.1 to 42.3 kg/m^2^ for Ghanaian women. In both cohorts, women in the first tertile were significantly younger and more likely to be nulliparous than those in the second and third tertiles. Indonesian women in the first tertile had more often a formal job than those in the second and third tertile, with unemployment being more common in the third tertile.

### Early pregnancy BMI, haemoglobin level and anaemia

**Fig. 1:**
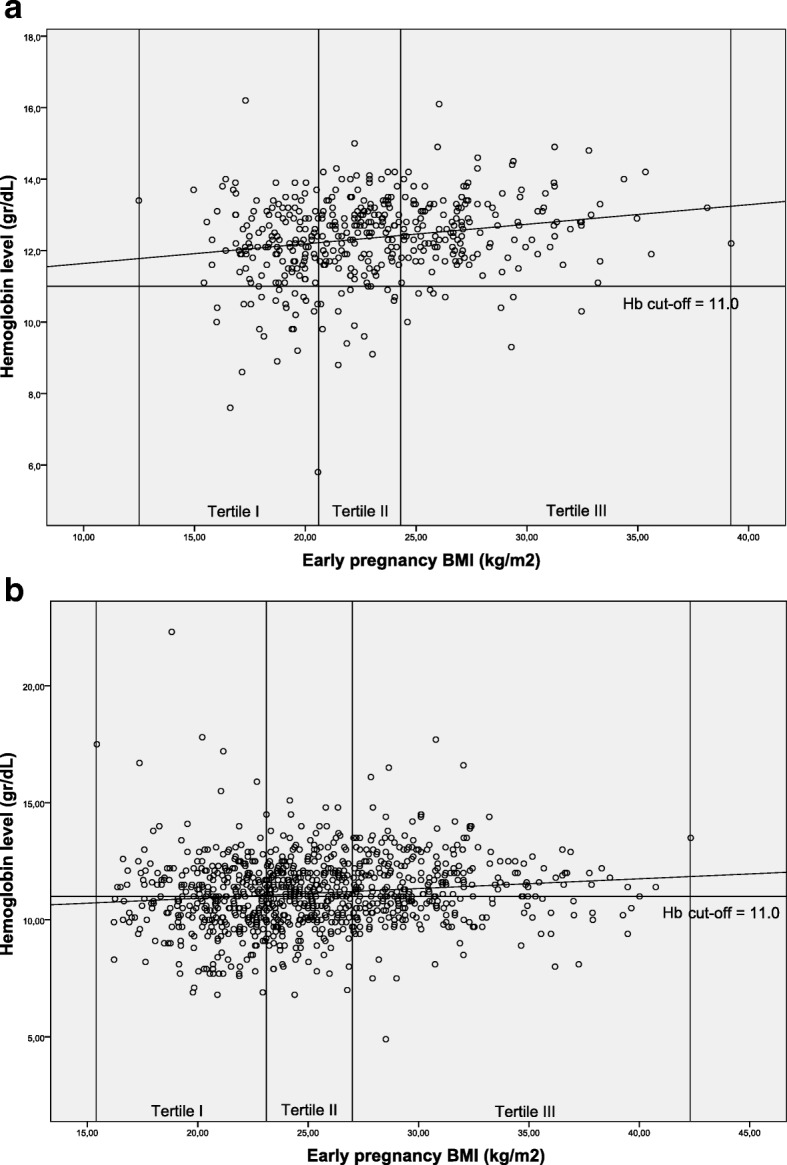
**a** and **b** Scatterplots of Indonesian and Ghanaian women divided into early pregnancy BMI groups by haemoglobin level; **a**: Indonesian cohort. **b** Ghanaian cohort

Mean BMI and the estimated prevalence of anaemia early in pregnancy were lower in Indonesian than in Ghanaian women (23.0 vs 25.4 kg/m^2^ and 10.4% vs 44.1%, respectively). Table 2 presents the association between early pregnancy weight, height and BMI, and risk of anaemia at first antenatal care visit. Haemoglobin concentration increased significantly with higher BMI in Indonesian (0.05 g/dL/kg/m^2^; 95% CI 0.03, 0.08) and Ghanaian women (0.04 g/dL/kg/m2; 95% CI 0.02, 0.07). Associations were unaltered by adjustment for potential confounders. As Fig. 1a and b illustrate, women with higher early pregnancy BMI tended to have higher haemoglobin levels. Correspondingly, higher early pregnancy BMI was associated with lower odds for anaemia at antenatal care booking in Indonesian (adjusted OR 0.88; 95% CI 0.81, 0.97) and Ghanaian women (adjusted OR 0.95; 95% CI 0.92, 0.98).

**Table 2:** Effect of early pregnancy weight (kg), height (m), and BMI (kg/m2) on haemoglobin concentration (g/dL) at booking with corresponding anaemia risks

		Indonesian cohort			Ghanaian cohort		
Linear regression							
		Coefficient	95% CI	*P*	Coefficient	95% CI	*P*
Weight (kg)							
Haemoglobin (g/dL)	Crude	0.021	0.01, 0.03	< 0.001	0.017	0.01, 0.03	< 0.001
	Model 1	0.02	0.01, 0.03	< 0.001	0.017	0.01, 0.03	< 0.001
	Model 2	0.02	0.01, 0.03	< 0.001	0.017	0.01, 0.03	< 0.001
	Model 3	0.02	0.01, 0.03	< 0.001	0.016	0.01, 0.02	< 0.001
Height (cm)							
Haemoglobin (g/dL)	Crude	−0.004	−0.02, 0.02	0.71	0.014	−0.001, 0.03	0.07
	Model 1	−0.004	−0.02, 0.02	0.67	0.012	− 0.003, 0.03	0.13
	Model 2	−0.005	−0.02, 0.01	0.62	0.012	−0.003, 0.03	0.13
	Model 3	−0.005	−0.02, 0.01	0.61	0.002	−0.01, 0.02	0.79
Early pregnancy BMI (kg/m2)							
Haemoglobin (g/dL)	Crude	0.054	0.03, 0.08	< 0.001	0.042	0.02, 0.06	< 0.001
	Model 1	0.054	0.03, 0.08	< 0.001	0.042	0.02, 0.06	< 0.001
	Model 2	0.054	0.03, 0.08	< 0.001	0.041	0.02, 0.06	< 0.001
	Model 3	0.054	0.03, 0.08	< 0.001	0.044	0.02, 0.07	< 0.001
Logistic regression							
		OR	95% CI	*P*	OR	95% CI	*P*
Weight (kg)							
Anaemia (< 11.0 g/dL)	Crude	0.97	0.94, 1.00	0.03	0.98	0.97, 0.99	< 0.001
	Model 1	0.96	0.93, 1.00	0.03	0.98	0.97, 0.99	0.001
	Model 2	0.96	0.93, 1.00	0.04	0.98	0.97, 0.99	0.001
	Model 3	0.96	0.93, 1.00	0.03	0.98	0.97, 0.99	0.002
Height (cm)							
Anaemia (< 11.0 g/dL)	Crude	1.04	0.99, 1.10	0.13	0.99	0.97, 1.01	0.53
	Model 1	1.05	0.99, 1.11	0.09	1	0.98, 1.02	0.75
	Model 2	1.05	0.99, 1.11	0.09	1	0.98, 1.02	0.75
	Model 3	1.05	0.99, 1.11	0.1	1.01	0.99, 1.03	0.5
Early pregnancy BMI (kg/m2)							
Anaemia (< 11.0 g/dL)	Crude	0.89	0.82, 0.97	0.01	0.95	0.92, 0.97	< 0.001
	Model 1	0.88	0.81, 0.97	0.01	0.95	0.92, 0.98	0.001
	Model 2	0.88	0.80, 0.97	0.01	0.95	0.92, 0.98	0.001
	Model 3	0.88	0.81, 0.97	0.01	0.95	0.92, 0.98	< 0.001

*OR* odds ratio

Results are expressed as linear regression coefficients of haemoglobin concentration in g/dL or logistic regression coefficients of maternal anemia for every 1 unit increase in exposures

Model 1 is adjusted for maternal age, education level and employment status

Model 2 is adjusted as for model 1 and for gravidity

Model 3 is adjusted as for model 2 and for secondhand smoking exposure in Indonesian cohort or anaemia-associated-comorbidities in Ghanaian cohort

Higher early pregnancy weight was found related with higher haemoglobin levels and lower risks for anaemia in both cohorts, but height was not.

### Anaemia and birth outcomes

The number of women with the composite adverse birth outcomes was 28 (6.1%) in Indonesia and 103 (10.9%) in Ghana. As is presented in Table 3, anaemia was not associated with adverse birth outcomes in neither the Indonesian (adjusted OR 1.29; 95% CI 0.35, 4.76) nor the Ghanaian (adjusted OR 1.09; 95% CI 0.71, 1.68) cohort.

**Table 3:** Crude and adjusted risks for the composite adverse birth outcomes according to maternal anaemia status at first booking

Logistic regression		Indonesian cohort			Ghanaian cohort		
		OR	95% CI	*P*	OR	95% C	*P*
Haemoglobin (g/dL)							
Adverse birth outcomes^a^	Crude	0.91	0.65, 1.27	0.58	0.97	0.85, 1.11	0.69
	Model 1	0.91	0.66, 1.27	0.59	0.97	0.85, 1.12	0.71
	Model 2	0.9	0.65, 1.26	0.54	0.98	0.85, 1.12	0.71
	Model 3	0.9	0.65, 1.25	0.53	0.98	0.85, 1.12	0.73
Anaemia (< 11.0 g/dL)							
Adverse birth outcomes^a^	Crude	1.4	0.40, 4.93	0.61	1.09	0.72, 1.67	0.68
	Model 1	1.26	0.35, 4.57	0.72	1.1	0.72, 1.69	0.66
	Model 2	1.3	0.36, 4.71	0.7	1.1	0.72, 1.69	0.66
	Model 3	1.29	0.35, 4.76	0.7	1.08	0.70, 1.69	0.71

OR, odds ratio ^a^Adverse birth outcomes included low birth weight, prematurity, and stillbirthResults are expressed as logistic regression coefficients of adverse birth outcomes with exposure categories

Model 1 is adjusted for maternal age, education level and employment status

Model 2 is adjusted as for model 1 and for gravidity

Model 3 is adjusted as for model 2 and for secondhand smoking exposure in Indonesian cohort or anemia-associated-comorbidities in Ghanaian cohort

## Discussion

This study showed that lower early pregnancy BMI is associated with lower haemoglobin levels and higher risk of anaemia at first antenatal care visit in Indonesian and Ghanaian women.

Estimated mean haemoglobin level in Indonesian pregnant women was 12.4 g/dL, which is slightly higher than the mean maternal haemoglobin of 11.7 g/dL reported previously by WHO [31]. The prevalence of maternal anaemia (10.4%) was much lower than in previous studies (prevalence estimates between 25 and 30%) [2, 31]. This could be due to the urban setting where the study was conducted, while previous studies included rural areas data. For the Ghanaian cohort, the estimated haemoglobin mean was comparable to that previously reported in Ghanaian and Sub-Saharan African pregnant women [2, 31].

Several studies reported that lower BMI in pre- or early pregnancy is associated with anaemia in pregnancy [14, 18, 36]. However, many had methodological limitations, including lack of adjustment for gestational age and differences in anaemia cut-off levels and gestational age at blood withdrawal [25, 37, 38]. These issues were addressed specifically in our study.

In both cohorts, higher early pregnancy weight was related to higher haemoglobin levels and lower risk for anaemia. Low early pregnancy weight or BMI may be a reflection of poor nutrition intake, including the intake of various micronutrients that are essential for haematopoiesis [1]. Furthermore, low weight or BMI could be a result of chronic illness, such as tuberculosis or parasitic infections, which consequently lead to anaemia [8, 25]. These conditions are still the major health problems globally and are often under-diagnosed [39–41].

Previous studies observed an association between anaemia in pregnancy and an increased risk of maternal mortality [11],adverse maternal outcomes including quality of life, and infant outcomes [1, 3]. In this study, we did not observe an association between early pregnancy anaemia and a composite of birth outcomes. We did not put BMI in the regression model because we considered BMI a mediator rather than a confounder. The absence of an association may be explained by the relatively low incidence of adverse events in both cohorts.

The high anaemia prevalence in Sub-Saharan Africa is attributed to factors such as poor nutrition, haemoglobinopathies, as sickle cell disease, or parasitic infections including malaria [2, 42]. Several studies suggest to consider lower haemoglobin cut-off values for diagnosis of anaemia in Sub-Saharan African populations [42, 43], as using universal cut-off for anaemia could result in a high number of anaemia cases not accompanied with significant clinical symptoms or increased risk of adverse pregnancy outcomes [12].

Both in Indonesia and Ghana, anaemia screening and treatment during antenatal care is recommended by (inter)national guidelines [44–46]. Potential intrapartum and postpartum complications associated with low haemoglobin levels, particularly postpartum haemorrhage, still in the top three causes of maternal mortality in Sub-Saharan Africa [10, 11, 47, 48], justify this recommendation. The extent to which anaemia screening is implemented in routine antenatal care, varies between countries [49]. Given the high prevalence of anaemia during pregnancy, intensifying routine screening (especially in the third trimester) and early initiation of treatment when routine supplementation of iron did not suffice, would be recommended.

Our study had several strengths. First, both cohorts have a prospective design and are conducted in similar time periods. This enables direct comparison between the two populations and minimizes possible cohort effect. Secondly, the strength of this study lies in its novelty. To the best of our knowledge, this is the first study which (simultaneously) investigated the association between early pregnancy BMI and maternal anaemia in Southeast Asia and Sub-Saharan Africa. These are the regions of the world with the highest prevalence of anaemia in pregnancy and the greatest anaemia related burden of disease [2, 3, 31]. The two cohorts had used similar terms of conduct and outcome measurement and had both a favourable low prevalence of red blood cell-affecting morbidities. Both cohorts also complemented each other in terms of subjects’ characteristics, which enabled comparison between the two settings and exploration of additional hypotheses. For example the influence of haemoglobin-affecting pathologies and secondhand smoking in the relation between women’s BMI and anaemia. In Jakarta, a malaria non-endemic area, malaria prevalence and haemoglobinopathies occurrence were very low [50, 51] in contrast to prevalence estimates in Accra, Ghana [52, 53]. Rate of second hand smoking exposure, on the other hand, are very low in Ghana (0.3%) compared to in Indonesia [28]. In addition, the characteristics of the cohort population support the generalisation of our findings to other urban low resource settings.

Certain points need to be considered in the interpretation of these findings. First, pre-pregnancy weight was asked to Indonesian women retrospectively and used as a proxy for early pregnancy weight. Consequently, inaccuracies may exist. Yet, when women’s pre-pregnancy weight was compared with their measured weight at antenatal booking, we found that they were highly correlated (R square > 0.85, data not shown). Therefore, pre-pregnancy weight was considered suitable as proxy for early pregnancy weight. Secondly, differences between Ghanaian and Indonesian women might exist regarding (gestational) age during enrolment and reported pre-existing hypertension. However, possible differences are minimal and were therefore considered inconsequential.

## Conclusions

In summary, in pregnant women from urban settings in Indonesia and Ghana, low early pregnancy BMI relates to an increased risk for anaemia at first antenatal care visit. Therefore, health providers should consider initiating early anaemia screening or treatment. Anaemia prevalence in Indonesians is notably lower than previously reported, whilst nearly four in ten Ghanaian women are anaemic according to international standards. Contrary to previous studies, in both cohorts anaemia was not associated with adverse birth outcomes.

## Additional file

Additional file 1: **Table S1.** Missing values upon baseline characteristics of the study populations. The supplementary table shows the incidence of missing data for each baseline characteristic. (XLSX 8 kb)

## Abbreviations

ANC: Antenatal care
BMI: Body mass index
G6PD: Glucose-6-phosphate dehydrogenase
GHS-ERC: Ghana Health Services Ethical Review Committee
LMIC’s: Low- and middle income countries
WHO: World Health Organization
